# Prevalence and Correlates of the Concurrence of Autism Spectrum Disorder and Obsessive Compulsive Disorder in Children and Adolescents: A Systematic Review and Meta-Analysis

**DOI:** 10.3390/brainsci14040379

**Published:** 2024-04-13

**Authors:** Claudia Aymerich, Malein Pacho, Ana Catalan, Noorulain Yousaf, Violeta Pérez-Rodríguez, Matthew J. Hollocks, Mara Parellada, Georgina Krebs, Bruce Clark, Gonzalo Salazar de Pablo

**Affiliations:** 1 Department of Child and Adolescent Psychiatry, Institute of Psychiatry, Psychology & Neuroscience, King’s College London, London WC2R 2LS, UK; 2 Biobizkaia Health Research Institute, Basque Country University, Basurto University Hospital, OSI Bilbao-Basurto, Centro de Investigación en Red de Salud Mental (CIBERSAM), Instituto de Salud Carlos III, 48903 Barakaldo, Spain; 3 Department of Psychosis Studies, Institute of Psychiatry, Psychology & Neuroscience, King’s College London, London WC2R 2LS, UK; 4 Child and Adolescent Mental Health Services, South London and Maudsley NHS Foundation Trust, London SE5 8AZ, UK; 5 Service for Complex Autism & Associated Neurodevelopmental Disorders, South London and Maudsley NHS Foundation Trust, London SE5 8AZ, UK; 6 Department of Child and Adolescent Psychiatry, Institute of Psychiatry and Mental Health, Hospital General Universitario Gregorio Marañón School of Medicine, Universidad Complutense, Instituto de Investigación Sanitaria Gregorio Marañón (IiSGM), Centro de Investigación en Red en Salud Mental (CIBERSAM), 28007 Madrid, Spain; 7 Research Department of Clinical, Educational and Health Psychology, University College London, London WC1E 6BT, UK; 8 National & Specialist OCD, BDD and Related Disorder Clinic, South London & Maudsley NHS Foundation Trust, London SE5 8AZ, UK

**Keywords:** obsessive compulsive disorder, autism spectrum disorder, compulsion, ritual, comorbidity

## Abstract

Background: Autism spectrum disorder (ASD) and obsessive compulsive disorder (OCD) are two common and impairing neurodevelopmental conditions with partial symptomatic overlap. The aim of this study is to systematically and meta-analytically examine the following: (i) the prevalence of an OCD diagnosis among young people with ASD, (ii) the prevalence of an ASD diagnosis among young people with OCD, and (iii) the clinical and therapeutic implications of such comorbidity. Method: A multistep literature search was performed from database inception until 17 November 2023. This PRISMA/MOOSE-compliant systematic review, registered in PROSPERO (CRD42023480543), identified studies reporting on the prevalence, sociodemographic, psychopathologic, prognostic, and therapeutic correlates of OCD and ASD concurrence in children and adolescents. A quantitative meta-analysis with random effects was conducted to analyse the pooled prevalence of OCD among samples with a mean age of < 18 years old with ASD and the prevalence of ASD among individuals under 18 with OCD. Sensitivity analyses were performed to investigate the effect of diagnostic criteria and different continents. Meta-regression analyses were conducted to examine the effect of gender, age, IQ, and OCD severity scores. A narrative review of the clinical and therapeutical implications of the comorbidity was provided. Results: 42 studies were selected for the systematic review (SR), and 31 of them were also included in one of the meta-analyses. The pooled prevalence of OCD among ASD youth samples (n = 8916, mean age = 10.6 ± 1.6; 16.4% female) was 11.6% (95% confidence intervals [CI] = 6.9%; 18.8%), and the pooled prevalence of ASD among OCD children and adolescent samples (n = 6209, mean age = 14.1 ± 1.4; 45.7% female) was 9.5% (95% CI = 6.0%; 14.7%). Meta-regressions found a statistically higher prevalence of ASD among samples with a lower prevalence of females (β = −4.7; 95%CI = −8.6; −0.8). Children with both OCD and ASD present higher rates of functional impairment, psychopathology, and other comorbidities, compared to youth with either of the disorders alone. Conclusions: OCD and ASD are highly concurrent conditions in youth, with symptomatic, prognostic, severity, and therapeutic implications. Future research should focus on conducting longitudinal cohort studies prospectively to determine development trajectories, along with randomized controlled trials to assess the efficacy of specific therapeutic interventions.

## 1. Introduction

Autism spectrum disorder (ASD) is a continuum of neurodevelopmental conditions characterized by impairments in reciprocal social communication and restricted, repetitive patterns of behaviour and interests [1]. Beyond the high burden of care associated with ASD, evidence reports extremely high levels of both physical and psychiatric comorbidity [2]. Up to 70% of children and adults with ASD meet the criteria for a comorbid psychiatric disorder, including but not limited to anxiety disorders, depressive and bipolar mood disorders, and schizophrenia spectrum disorders [3,4,5].

One disorder that frequently co-occurs among individuals with ASD is obsessive compulsive disorder (OCD), a common condition characterized by unwanted, recurrent thoughts (obsessions) and time-consuming, repetitive behaviours (compulsions), which cause clinically significant distress and/or substantial functional impairment [6]. Neurodevelopmental, mood, and anxiety comorbidities are also commonly found among individuals with OCD across the lifespan [7].

Many individuals with ASD exhibit a heterogeneous set of behaviours, that partially overlap with symptoms associated with OCD: routine fixation, ritualized patterns of behaviour, resistance to change, and highly selective interests, among others [8]. ASD-related repetitive behaviours can serve different functions in autism, such as self-stimulation, anxiety reduction, or enjoyment, and are usually ego-syntonic [9,10,11]. In OCD, compulsions tend to be ego-dystonic and seem to be anxiety driven and anxiety-neutralizing [12]. However, this can be difficult to ascertain in younger children, especially when language and communication are impaired [13], meaning that symptoms can be challenging to differentiate. In addition, many individuals meet criteria for both disorders and consequently may require a differential therapeutic approach to ascertain the nuclear dysfunction behaviours. Moreover, studies suggest that people with co-occurring ASD and OCD may present different clinical characteristics and symptom expressions than those with either of the diagnoses alone. Furthermore, it has been suggested that dual diagnosis involves greater severity, functional impairment, and a poorer therapeutic response to therapeutic interventions (including both pharmacological and psychotherapeutic approaches), compared to samples with either only OCD or only ASD [13,14,15]. 

Although a few meta-analyses have previously examined the extent and the implications of the co-occurrence of ASD and OCD across the lifespan, they have done so with important limitations. The rate of co-occurrence remains highly variable, ranging from 6% to 37% [16,17], and paediatric samples are often under-represented compared to adults [7]. Furthermore, restrictive criteria, such as the exclusion of all experimental studies, limit the external validity of their findings [3]. Indeed, findings have been inconsistent in terms of prevalence, clinical, and therapeutic correlates. No study so far has meta-analytically examined the prevalence of the comorbidity of ASD and OCD in children and adolescents, and its implications in terms of psychopathology, prognosis, and therapeutic implications.

To fill this gap, this systematic review and meta-analysis aims to examine the following: (i) the prevalence of an OCD diagnosis among individuals with ASD, (ii) the prevalence of an ASD diagnosis among individuals with OCD, and (iii) the sociodemographic, psychopathological, prognostic, and therapeutic correlates of such comorbidity.

## 2. Methods

This study protocol was registered on PROSPERO (registration number: CRD42023480543). The study was conducted in accordance with “Preferred Reporting Items for Systematic Reviews and Meta-Analyses” (PRISMA) [18] (Table S1) and “Meta-Analyses of Observational Studies in Epidemiology” (MOOSE) checklists [19] (Table S2), following “EQUATOR Reporting Guidelines” [20].

### 2.1. Search Strategy and Selection Criteria

Two independent researchers (Aymerich and Pacho) conducted a systematic search of the literature, up until 17 November 2023. The searches were performed using the Web of Science database (Clarivate Analytics, London, UK), incorporating the Web of Science Core Collection, the BIOSIS Citation Index, the KCI-Korean Journal Database, MEDLINE^®^, the Russian Science Citation Index, the SciELO Citation Index, the Cochrane Central Register of Reviews, and Ovid/PsycINFO databases.

The following keywords were used: (“obsessive compulsive disorder” OR “obsessive-compulsive” OR “OCD” OR “obsess*” OR “compuls*”) AND (“autism” OR “autistic” OR “child development* disorder” OR “pervasive development* disorder*” OR “Asperger” OR “Asperger’s syndrome” OR “childhood disintegrative disorder”) AND (“comorbid*” OR “coexist*” OR “association” OR “relationship” OR “interlink” OR “overlap” OR “co-occur*”).

The inclusion criteria for the systematic review were as follows: (a) individual studies with original data, (b) conducted in children and adolescents (mean age < 18 years), (c) reporting on a sample meeting criteria for ASD (including pervasive developmental disorder) or OCD, according to DSM criteria (any version) [1,21,22] or ICD criteria (any version) [23,24], (d) evaluating the prevalence, associated factors, clinical, or therapeutical implications, of the comorbidity between OCD and ASD disorders, and (e) written in English. Additional criteria for the meta-analyses included the following: (f) samples where the presence of concurrent and/or comorbid disorders is formally assessed by validated scales or a clinical assessment and (g) non-overlapping samples. When multiple data points were available in one study, the latest point recorded was coded. Studies were examined for samples overlap, determined by looking at the inclusion dates and type of population and country in which the study was carried out; in case of overlapping samples, the study with the largest sample was then selected.

The exclusion criteria consisted of the following: (a) reviews, editorials, conference proceedings, or single case reports, (b) reporting on individuals not meeting DSM or ICD diagnostic criteria for the aforementioned disorders (e.g., diagnosed by self-reported scales), (c) written in languages other than English, and (d) reporting on samples with fewer than 20 individuals. This threshold was chosen in accordance with previous studies on the field [3], with the aim of maximizing the available information while minimizing selection bias. 

Identified articles were first screened by the two independent reviewers as abstracts, and after excluding those not meeting the inclusion criteria, the full texts of the remaining articles were assessed for eligibility. The process resulted in an overall interrater agreement of 93%. In cases of disagreement, a senior researcher (G.S.P.) made the final decision. The search was completed by manually searching through references of previously published systematic reviews and meta-analyses on the topic.

### 2.2. Data Extraction

Two researchers (Pacho and Yousaf) independently extracted data from all the included studies. The databases were then cross-checked by an independent researcher (Aymerich), and discrepancies were resolved by consensus. The selected variables included the following: first author and year of publication, country, recruiting period, study type (cross-sectionals, cohorts, case–control, clinical trial), sample size, diagnostic criteria, age (mean ± standard deviation [SD] and age range), percent of females, intelligence quotient (IQ) (mean ± SD), symptom severity, study quality (see below), and key findings.

### 2.3. Risk of Bias (Quality) Assessment

Risk of bias was assessed using a modified version of the Newcastle–Ottawa Scale (NOS) for cross-sectional and cohort studies. Studies were awarded 0–9 points, according to their representativeness, exposure, outcomes, follow-up period, and losses to follow-up (Table S3). Scores of ≥7–9, 4–6, and <4 were considered to have a low, intermediate, and high risk of bias, respectively [25].

### 2.4. Strategy for Data Synthesis and Statistics

A systematic synthesis of the findings from the included studies was provided. The available evidence was structured in two tables; one included all the studies studying the prevalence and/or the clinical and therapeutic correlates of OCD among ASD samples (Table 1), and the other included articles on the prevalence and/or the clinical and therapeutic correlates of ASD among OCD samples (Table 2). When an article reported on samples with dual diagnoses, a decision on what table to include the article in was made according to the primary focus of each study. A narrative review was provided, enumerating the sociodemographic and prognostic factors, psychopathological features, and therapeutic correlates associated with ASD and OCD concurrence in children and adolescents.

Then, we performed two separate analyses. First, we performed a meta-analysis using, as the primary effect size, the proportion (% and standard error [SE], when available) of OCD comorbidity among ASD samples. Second, the proportion (% and SE) of ASD comorbidity among OCD samples was meta-analysed.

Meta-regressions were performed when a minimum of seven papers were available to study the effects of (a) the mean age of the sample, (b) the % of females, (c) the mean IQ of the sample, (d) the Children’s Yale–Brown Obsessive Compulsive Scale (CY-BOCS) severity score of the sample, (e) the Newcastle–Ottawa Scale (NOS) score, and (f) the year of publication. Sensitivity analyses were performed to study the influence of the diagnostic criteria (ICD versus DSM), ASD sample type (including individuals with PDD versus only including individuals with ASD), and the continent of origin of the sample, when more than seven articles were available. Heterogeneity among studies was assessed using Q statistics, with the proportion of the total variability in effect size estimates evaluated using the I^2^ index, classifying the heterogeneity as low (I^2^ = 25%), medium (I^2^ = 50%), and high (I^2^ = 75%) [26]. Since heterogeneity was expected to be high, the random effect model was used. Publication bias was assessed by visually inspecting funnel plots and performing Egger’s test, when more than seven articles were available for a particular outcome [27].

All analyses were conducted within R software, version 1.4.1106, using the metaprop function with the package ‘meta’ [28]. The significance level was set at *p* < 0.05, two-sided.

**Table 1:** Articles reporting on samples of children and adolescent with autism spectrum disorder.

Author (Year)	City (Country)	NOS Score	Sample Size	ASD Diagnostic Criteria	Setting	Sample Type	Mean IQ ± SD	Mean Age ± SD (Range)	Females (%)	Ethnicity (%)	Key Findings
Amr (2012) * [29]	Multiple (Jordan, Egypt, and Saudi Arabia)	6	60	DSM-IV-TR	Clinical	AS, PDD-NOS	61.3 ± 21.6	8.4 ± 1.8 (6–11 years)	38.3	N.a.	Of individuals with ASD, 55.0% met criteria for OCD (DSM-IV-TR). OCD was the most prevalent comorbid disorder among the ASD sample.
Gurkan (2009) * [30]	Ankara (Turkey)	5	40	DSM-IV-TR	Clinical	ASD (N = 18), AS (N = 11), PDD-NOS (N = 11)	88.5 ± 18.9	10.8 ± 3.6 (6–18 years)	10.0	N.a.	Of the individuals with ASD, 15.8% met criteria for OCD (DSM-IV-TR).
Leyfer (2006) * [31]	Boston, Salt Lake City (USA)	8	109	DSM-IV-TR	Community	ASD with some spoken language	81.5 ± 24.5	9.2 ± 2.7 (5–17 years)	5.7	N.a.	Of the individuals with ASD, 37.2% met criteria for OCD (DSM-IV-TR). The most common type of compulsion was a ritual involving other individuals having to perform a certain way (e.g., greeting and separation rituals, question-asking rituals).
Margari (2019) * [32]	Bari (Italy)	7	159	DSM-5	Clinical	High functioning ASD	106.5 ± 16.9	10.3 ± 4.3 (N.a.)	37.1	N.a.	Of the individuals with ASD, 5.0% of male individuals and 0.0% of female individuals met criteria for OCD (DSM-5).
Mazefsky (2012) * [33]	Pittsburgh (USA)	6	35	DSM-IV	Community/Clinical	High functioning ASD	105.0 ± 17.0	12.1 ± 2.0 (10–17 years)	20.0	Caucasian (88.6)	When a structured psychiatric interview carefully took ASD-related impairment into account using the autism comorbidity interview, only 2.9% of the individuals with ASD met criteria for OCD (DSM-IV-TR)
Sturm (2004) * [34]	Stockholm (Sweden)	6	101	DSM-IV	Clinical	AS (N = 91), PDD-NOS (N = 9), high functioning ASD (N = 1)	N.a.	9.8 ± N.a. (5–12 years)	29.7	N.a.	Of the individuals with ASD, 6.9% met criteria for OCD (DSM-IV). Also, 48.5% children had symptoms of obsessive compulsive disorder, and, in 24.8%, these symptoms were severe.
Fucà (2023) * [35]	Rome (Italy)	6	472	DSM-5	Clinical	ASD with IQ > 70	90.6 ± 19.3	7.2 ± 3.4 (3–18 years)	17.5	N.a.	Of the individuals with ASD, 0.6% met criteria for OCD (DSM-5). Adolescents exhibited greater prevalence of OCD and anxiety disorders than preschoolers.
Gjevik (2011) * [36]	Oslo (Norway)	7	71	DSM-IV	Community	AS (N = 12), PDD-NOS (N = 12), ASD (N = 47)	65.2 ± 29.6	11.8 ± 3.3 (6–18 years)	18.3	N.a.	Of the individuals with ASD, 9.9% met criteria for OCD (DSM-IV criteria). OCD was more common in older children. Children with OCD had worries about contamination, checking, and hand washing.
Joshi (2010) * [37]	Boston (USA)	8	217	DSM-III-R	Clinical	ASD/PDD-NOS	N.a.	9.7 ± 3.6 (3–17.9 years)	13.4	N.a.	Of the individuals with ASD, 24.4% met criteria for OCD (DSM-III-R).
Romero (2016) * [38]	Málaga (Spain)	6	123	DSM-IV-TR/DSM-5	Community	ASD (N = 57), PDD-NOS (N = 66)	N.a.	10.6 ± 3.0 (5–15.9 years)	18.0	N.a.	Of the individuals with ASD, 40.7% met criteria for OCD (DSM-5). Patients fulfilling criteria for DSM-5 criteria for ASD had higher prevalences of OCD.
Van der Plas (2016) * [17]	Toronto (Canada)	7	99	DSM-IV	Community	ASD	N.a.	11.0 ± 2.3 (6–17.9 years)	12.1	Caucasian (29.3)	Of the individuals with ASD, 6.2% met criteria for OCD (DSM-IV). Among those that did not meet criteria, ASD youth exhibited more OCD traits than individuals without ASD.
Caamaño (2013) * [39]	Madrid (Spain)	7	25	DSM-IV-TR	Clinical	ASD without intellectual disability	97.9 ± 27.6	12.8 ± 2.9 (7–17.9 years)	4.0	Caucasian (92.0)	Of the individuals with ASD, 12.5% met criteria for OCD (DSM-IV-TR). ASD youth exhibited more OCD traits than individuals without ASD, even in the absence of a concurrent disorder.
Muris (1998) * [40]	South-Limburg (The Netherlands)	7	44	DSM-III-R	Clinical	ASD (N = 15), PDD-NOS (N = 29)	79.5 ± 14.0	9.7 ± 4.8 (2–17.9 years)	N.a.	N.a.	Of the individuals with ASD, 11.4% met criteria for OCD (DSM-III-TR). An additional 61.3% of the sample also exhibited rituals, but most parents or guardians did not know whether these rituals caused distress.
Simonoff (2008) * [41]	Multiple (UK)	8	112	ICD-10	Community	ASD (N = 62), PDD (N = 50)	72.7 ± 21.6	11.5 ± N.a. (10–14.9 years)	12.5	White (95.0)	Of the individuals with ASD, 8.2% met criteria for OCD (DSM-IV). The authors suggested their rates were lower than other studies because they required a purposeful quality to obsessional thoughts and compulsions in order to meet criteria.
de Bruin (2006) * [42]	Rotterdam (The Netherlands)	8	94	DSM-IV	Clinical	PDD-NOS	91.2 ± 17.4	8.5 ± 1.9 (6–12.9 years)	11.7	N.a.	Of the individuals with ASD, 6.4% met criteria for OCD (DSM-IV).
Green (2000) * [43]	Manchester (UK)	5	20	ICD-10	Clinical	AS	92.2 ± 17.7	13.8 ± N.a. (11–19 years)	0.0	N.a.	Of the individuals with ASD, 25.0% met criteria for OCD (ICD-10). The authors noted the difficulty in distinguishing between obsessive compulsive symptoms and the bizarre preoccupations characteristic of the core autistic disorder.
Mattila (2010) * [44]	Multiple (Finland)	7	50	DSM-IV	Community/Clinical	AS	>75	12.7 ± 1.5 (9.8–16.3 years)	24.0	N.a.	Of the individuals with ASD, 22.0% met criteria for OCD (DSM-IV).
Mukaddes (2010) * [45]	Istanbul (Turkey)	7	60	DSM-IV	Clinical	AS (N = 30), HF-ASD (N = 30)	98.5 ± N.a.	10.7 ± N.a. (6–15.9 years)	0.0	N.a.	Of the individuals with ASD, 37.2% met criteria for OCD (DSM-IV). No significant differences were found between AS and HF-ASD in the prevalence of this co-occurrence.
Witwer (2010) * [46]	Ohio (USA)	6	61	DSM-IV	Clinical	AS (N = 16), ASD (N = 17), PDD-NOS (N = 26)	68.4 ± 23.3	11.2 ± 3.8 (6–17.9 years)	82.0	Caucasian (77.0)	Of the individuals with ASD, 4.9% met criteria for OCD (DSM-IV). However, an additional 42.6% reported significant OCD symptoms without meeting criteria. OCD symptom endorsement was not impacted by IQ. The authors noted the difficulty in distinguishing OCD symptoms from ASD-related repetitive behaviours.
Wozniak (1997) * [47]	Massachusetts (USA)	6	52	DSM-III-R	Clinical	PDD	N.a.	9.8 ± 3.8 (Range N.a.)	24.0	N.a.	Of the individuals with ASD, 16.0% met criteria for OCD (DSM-III-R).
King (2009) [48]	Multiple (USA)	8	149	DSM-IV-TR	Clinical	ASD or AS with >8 score for compulsive behaviours on CY-BOCS	N.a.	9.1 ± 3.2 (5–17 years)	16.8	Caucasian (72.5), Black (11.4), other (16.1)	Citalopram was not superior to placebo (*p* = 0.81) in the treatment of compulsive behaviour in children and adolescents with ASD.
Iniesta-Sepúlveda (2017) [49]	Multiple (USA)	5	9	DSM-IV-TR	Clinical	Primary OCD + high functioning ASD	N.a.	14.0 ± 2.0 (11–17 years)	11.1	Caucasian (89.0), Hispanic (11.0)	Intensive (3–6 h/day, 24–80 sessions) CBT with ASD-specific modifications was effective for 78% of participants, and large treatment effects (*d* = 1.35–2.58) were obtained in OCD symptom severity.
Mandell (2008) [50]	Pennsylvania (USA)	6	760	DSM	Community	ASD, AS, PDD-NOS	N.a.	9.3 ± 4.3 (5–21 years)	16.0	White (83.0), African American (8.9)	Concurrent OCD was a significant risk factor for psychiatric hospitalization among children with ASD (OR 2.35; *p* < 0.01).

Studies marked with an asterisk (*) were included in the meta-analyses. AS: Asperger’s syndrome; ASD: autism spectrum disorder; DSM: Diagnostic and Statistical Manual of Mental Disorders; HF-ASD: high functioning autism spectrum disorder; ICD: International Classification of Diseases; IQ: intelligence quotient; NOS: Newcastle–Ottawa Scale; OCD: obsessive compulsive disorder; OR: Odds Ratio; PDD-NOS: pervasive developmental disorder not otherwise specified; SD: standard deviation.

**Table 2:** Articles reporting on samples of children and adolescents with obsessive compulsive disorder.

Author (Year)	City (Country)	NOS Score	Sample Size	OCD Diagnostic Criteria	Setting	OCD Severity	Mean Age ± SD (Range)	Females (%)	Ethnicity (%)	Key Findings
Rintala (2017) * [51]	Multiple (Finland)	8	3372	ICD-9; ICD-10	Clinical	N.a.	15.2 ± 4.1 (3–25 years)	53.2	N.a.	Of individuals with OCD, 13.4% of male individuals and 3.4% of female individuals met criteria for an ASD disorder (ICD-9 299X; ICD-10 F84). Females were significantly less likely to present this co-occurrence (OR 0.22; *p* < 0.01).
Schachar (2022) * [52]	Ontario (Canada)	6	171	DSM-5	Clinical/Community	TOCS 65.4 ± 9.2	12.7 ± 2.6 (7–17.9 years)	40.4	N.a.	Of individuals with OCD, 8.8% of male and 4.3% of female individuals with OCD met criteria for an ASD disorder (DSM-5, ADI-R, ADOS-2). Antipsychotic use was more common among OCD cases with ASD.
Griffiths (2017-A) * [53]	Southport (Australia)	7	80	DSM-IV	Community	CY-BOCS 25.7 ± 4.6	12.3 ± 2.7 (7–17.9 years)	55.0	White (90.0), Black (2.5), Native Asian (2.5)	Of the individuals with OCD, 5.0% met criteria for an ASD disorder. An additional 27.5% of the sample showed moderate ASD traits. ASD traits were associated with greater functional impairment above OCD severity. Family accommodation mediated the relationship between ASD traits and functional impairment.
Farrell (2012) * [54]	Southport (Australia)	6	43	DSM-IV	Clinical	CY-BOCS 21.4 ± 6.6	11.1 ± 2.5 (7–17.9 years)	30.2	N.a.	Of the individuals with OCD in the sample, 34.9% met criteria for a PDD disorder. This did not significantly influence treatment response to a group CBT intervention at post-treatment but was associated with poorer treatment response at a 6-month follow-up.
Arildskov (2015) * [55]	Multiple (Denmark, Norway, and Sweden)	7	257	DSM-IV-TR	Clinical	CY-BOCS 24.7 ± 5.1	12.8 ± 2.8 (7–17.9 years)	51.4	N.a.	Of the individuals with OCD, 9.7% met criteria for an ASD disorder, with a male preponderance with a sex ratio of 2.6:1. Autism-specific social and communication difficulties were not related to OCD severity, while restricted repetitive behaviour was positively related to OCD severity.
Ivarsson (2007) * [56]	Gothenburg (Sweden)	8	109	DSM-IV	Clinical	N.a.	N.a.	55.0	N.a.	While only 8.3% of the individuals with OCD met criteria for an ASD disorder, autistic traits were common among the sample. OCD symptom scores were highest in cases with concurrent ASD.
Martin (2020) * [57]	London (UK)	8	1345	ICD-10	Clinical	N.a.	14.0 ± 2.6 (4–17.9 years)	52.2	White (66.2), Black (6.2), Asian (4.9)	Of the individuals with OCD, 24.9% met criteria for an ASD disorder. Youth with OCD + ASD had lower psychosocial functioning compared to those with either OCD or ASD, and showed significant improvements in functioning after service utilization, but their gains were smaller than those with OCD.
Jaspers-Fayers (2017) * [58]	Vancouver (Canada)	6	136	DSM-IV	Clinical	CY-BOCS 21.2 ± 8.0	13.1 ± 2.7 (6–19 years)	46.0	White (71.0), Asian (18.0), other (11.0)	Of the individuals with OCD, 6.0% met criteria for a PDD.
Adam (2019) * [59]	Cologne (Germany)	7	181	ICD-10	Clinical	N.a.	13.2 ± 3.0 (6–18 years)	49.7	N.a.	Of the individuals with OCD, 5.0% met criteria for a PDD.
Sevilla-Carmeno (2019) * [60]	Stockholm (Sweden)	7	193	ICD-10/DSM-5	Clinical	CY-BOCS 22.6 ± 4.4	13.6 ± 2.4 (6–17 years)	55.4	N.a.	Of the individuals with OCD, 19.7% met criteria for ASD.
Peris (2017) * [61]	Los Angeles (USA)	6	322	DSM-IV-TR	Clinical	CY-BOCS 25.2 ± 4.8	12.3 ± 2.8 (7–17 years)	47.0	White (72.0), Hispanic (10.0)	Of the individuals with OCD, 3.0% met criteria for ASD.
Griffiths (2017-B) [62]	Southport (Australia)	7	50	DSM-IV	Community	CY-BOCS 23.3 ± 6.6	12.0 ± 2.5 (7–17 years)	12.0	N.a.	Concurrent OCD and ASD was associated with significantly higher functional impairment (*t* = 2.91; *p* < 0.01), and more concurrent disorders overall (*X*^2^ = 2.51; *p* = 0.02) than OCD without concurrent ASD. Families of individuals with concurrent OCD and ASD engaged in more accommodating behaviours (*t* = 3.41; *p* < 0.01). Youth with both disorders had poorer treatment response (*t* = 7.67; *p* < 0.01) at a six month follow-up.
Lewin (2011) [63]	Multiple (USA)	7	70	DSM-IV	Clinical	CY-BOCS 26.2 ± 7.2	9.9 ± 1.8 (7–13 years)	20.0	Caucasian (74.0), Hispanic (4.0), other (22.0)	Individuals with OCD and concurrent ASD did not differ from patients with OCD for total severity scores, obsessions, or compulsions scores. ADHD symptoms, social phobia, and separation anxiety disorder were more common among youth with ASD + OCD. Youth with ASD + OCD were significantly less likely to endorse sexual obsessions, checking, or washing or repeating compulsions.
Murray (2015) [64]	London (UK)	8	44	ICD-10	Clinical	CY-BOCS 29.4 ± 5.0	15.0 ± 3.8 (7–13 years)	40.9	N.a.	Individuals with OCD and concurrent ASD (N = 22), compared to age-matched OCD youth without ASD (N = 22) showed lower rates of response (38.3% vs. 48.2%) and remission (9.0% vs. 46.0%) to CBT.
Wickberg (2022) [65]	Stockholm (Sweden)	6	22	DSM-5	Clinical	CY-BOCS 21.7 ± 3.7	13.9 ± 1.6 (7–17 years)	33.3	N.a.	Internet-delivered CBT was deemed acceptable and was associated with clinically significant improvement in CY-BOCS scores with a large within-group effect-size (*d* = 1.33). However, in-person CBT produced significantly larger effects (*d* = 2.69) among the same population.
Jassi (2021) [66]	London (UK)	7	34	ICD-11	Clinical	CY-BOCS 27.7 ± 4.2	15.2 ± 1.7 (7–17 years)	32.4	N.a.	An autism-adapted CBT manual for adolescents with ASD was found to present significant rates of response (52.9%) and remission (35.3%) that maintained at a 3-month follow-up in a small, naturalistic study.
Jassi (2023) [67]	London (UK)	9	619	ICD-11	Clinical	CY-BOCS 27.7 ± 4.9	14.6 ± 2.2 (6–18 years)	46.8	N.a.	Young individuals with OCD + ASD (N = 172) were more likely to endorse poorer insight into their OCD (*t* = −2.56; *p* < 0.01), have greater functional impairment (CGAS score *t* = 4.10; *p* < 0.01), greater levels of concurrent psychopathology (SDQ total difficulties score *t* = −6.27; *p* < 0.01), higher levels of family accommodation (*t* = −4.32; *p* < 0.01), and were more likely to be on medication (*X*^2^ = 22.6; *p* < 0.01), compared to OCD youth without concurrent ASD (N = 447). Whilst both groups benefitted from CBT ± medication, the OCD–ASD group had significantly poorer treatment responses (OR = 0.34; *p* < 0.01).
Højgaard (2023) [68]	Multiple (Denmark, Norway, and Sweden)	7	257	DSM-IV	Community	CY-BOCS 24.7 ± 5.1	12.8 ± 2.8 (7–17 years)	51.4	N.a.	Of the individuals with OCD, 9.70% presented clinically significant autistic traits (as measured with ASSQ, cut-off 17). Comorbid ADHD (OR 7.06) and tic disorders (OR 7.11), subclinical internalizing and externalizing symptoms according to CBCL (OR from 1.18 to 1.58), low insight (OR 1.79), and ordering/arranging OCD symptoms (OR N.a., *p* < 0.01) were found to be significantly associated with autistic traits. Individuals with OCD with or without autistic traits did not differ on CBT treatment outcomes.
Vause (2015) [69]	Ontario (Canada)	7	14	DSM-5	Clinical	CY-BOCS compulsions 14.6 ± 1.6	9.7 ± 1.5 (8–12 years)	35.7	N.a.	In a preliminary RCT, a combination of traditional CBT and function-based behavioural assessment significantly decreased OCD-like behaviours at post-treatment and a 5-month follow-up (F = 6.98; *p* = 0.02).
Mack (2010) [70]	London (UK)	6	24	ICD-10	Clinical	CY-BOCS 24.0 ± 6.8	14.3 ± 1.7 (12–18 years)	16.7	N.a.	Individuals with OCD only did not present statistically significant differences from individuals with co-occurrent OCD and ASD. However, individuals meeting criteria for both diagnoses reported more peer relationship problems.

Studies marked with an asterisk (*) were included in the meta-analyses. ASSQ: Autism Spectrum Screening Questionnaire; AS: Asperger’s syndrome; ASD: autism spectrum disorder; CBCL: Child Behaviour Checklist; CBT: cognitive behavioural therapy; CY-BOCS: Children’s Yale–Brown Obsessive Compulsive Scale; DSM: Diagnostic and Statistical Manual of Mental Disorders; ICD: International Classification of Diseases; HF-ASD: high functioning autism spectrum disorder; IQ: intelligence quotient; NOS: Newcastle–Ottawa Scale; OCD: obsessive compulsive disorder; PDD-NOS: pervasive developmental disorder not otherwise specified; RCT: randomized controlled trial; SD: standard deviation.

## 3. Results

**Figure 1:**
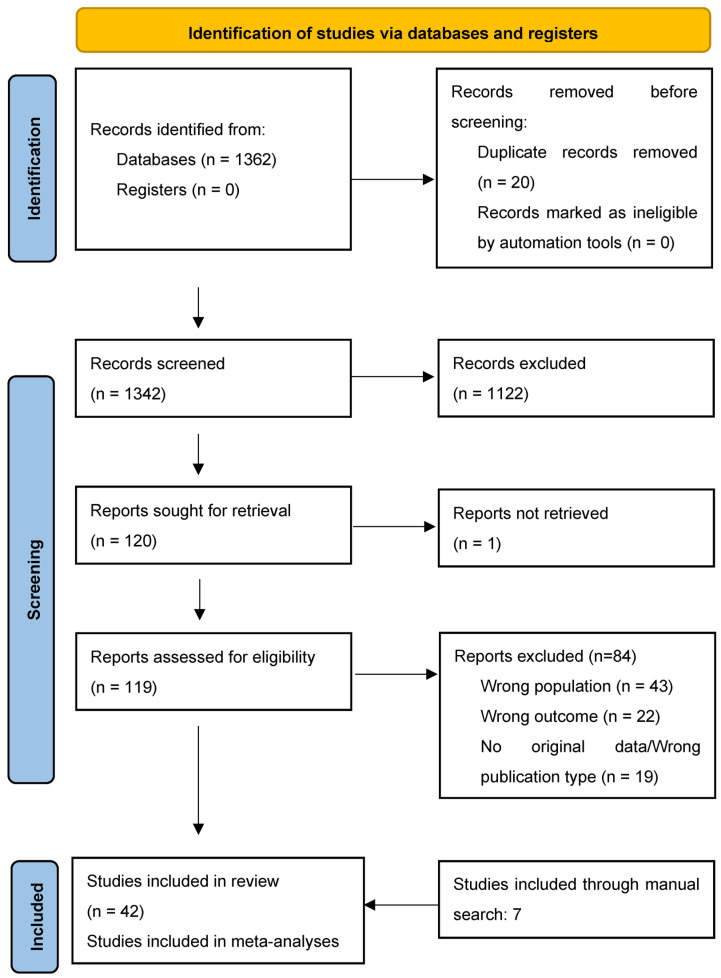
PRISMA 2020 flow diagram [71].

The literature search yielded 1342 citations through electronic databases, which were screened for eligibility; the full text of 119 articles were assessed, and 84 were excluded. Seven additional studies were included through manual search. The final database for the systematic review included 42 studies, of which 31 were also included in the meta-analyses, as can be seen in Figure 1 [71]. Studies included samples from 17 countries in five continents, mainly Europe (k = 24; 57.1%) and North America (k = 14; 33.3%).

### 3.1. Prevalence of Obsessive Compulsive Disorder among Children and Adolescents with Autism Spectrum Disorder

Data were extracted from 21 studies with a total sample size of 8916 patients (mean age = 10.58 ± 1.59 years old; 16.40% female). The mean IQ of the sample was 85.64 ± 14.58. Of the studies, 52.38% reported exclusively on samples of children and adolescents with a diagnosis of ASD, while 47.62% of the studies also included individuals with a pervasive developmental disorder (PDD) diagnosis. All studies included outpatient samples, and 93.7% of the sample was sourced through clinical settings (Table 1).

**Figure 2:**
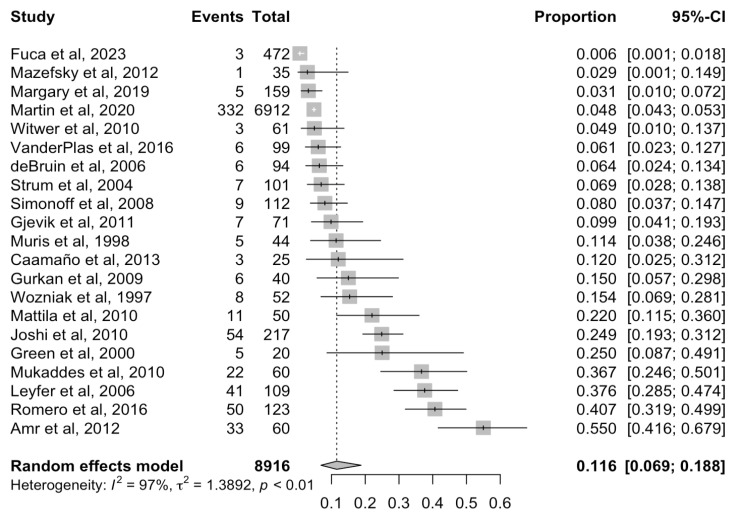
Forest plot for the prevalence of obsessive compulsive disorder among children and adolescents with autism spectrum disorder. CI: confidence interval.

The pooled prevalence of children and adolescents with ASD meeting ICD/DSM criteria for an OCD diagnosis was 11.55% (95% confidence intervals [CI] 6.88–18.76%) (Figure 2). High heterogeneity was detected (Q = 540.82; I^2^ = 96.6%; *p* < 0.05). 

Meta-regressions found no statistically significant effects of age, sex, IQ mean, risk bias, or publication year (all *p* > 0.05) (Table S4). Sensitivity analyses revealed no significant effect of the diagnostic criteria used (ICD vs. DSM) (Table S5). Publication bias was not identified neither by visual inspection of the funnel plot (Figure S1) nor by Egger’s test (*p* = 0.09) [22,23,24,25,26].

### 3.2. Prevalence of Autism Spectrum Disorder among Children and Adolescents with Obsessive Compulsive Disorder

Data were extracted from 11 studies for a total sample size of 6209 patients (mean age 14.1 ± 1.4; 45.7% female). The mean severity score for the CY-BOCS of the sample was 23.9 ± 5.4, and 98.7% of the sample was sourced through clinical settings (Table 2).

**Figure 3:**
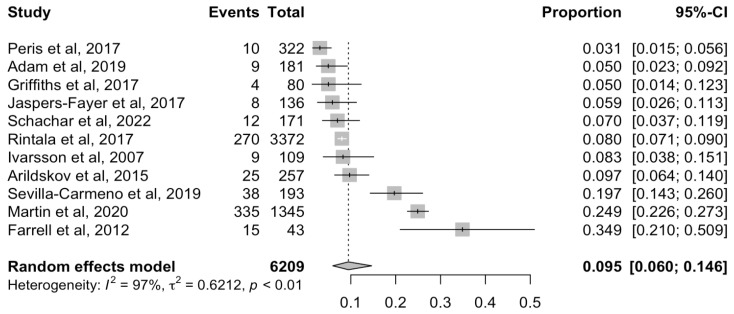
Forest plot for the prevalence of autism spectrum disorder among children and adolescents with obsessive compulsive disorder. CI Confidence Interval.

The pooled prevalence of children and adolescents meeting ICD/DSM criteria for an ASD diagnosis was 9.46% (95% CI 5.98–14.65%) (Figure 3). Meta-regressions found a statistically significant higher prevalence of ASD among samples with a lower percentage of female individuals (β = −4.69; 95% CI −8.59; −0.79), but no statistically significant effect of age, risk bias, or year of publication year (Table S6). High heterogeneity was detected (Q = 305.21; I^2^ = 96.7%; *p* < 0.05). Publication bias was not identified, neither by visual inspection of the funnel plot (Figure S2) nor by Egger’s test (*p* = 0.40).

### 3.3. Sociodemographic Factors

In addition to the meta-regressions performed in our analyses, several studies suggested potential sociodemographic factors related to the comorbidity of OCD and ASD. Margari et al. found OCD to be more prevalent among male than female children with ASD [32]. Consistently, three studies found ASD criteria more likely to be met in male children with OCD than in female children with the same disorder, with an odds ratio (OR) between 2.6 and 4.54 [51,52]. Two additional studies suggested older age to be a risk factor for OCD among ASD samples, with higher rates of OCD among older children (*p* < 0.001) and in adolescents than in preschoolers and toddlers (F = 6.11; *p* = 0.03) [35,36].

### 3.4. Psychopathological Features 

Three studies suggested psychopathological correlates on the OCD and ASD comorbidity in terms of symptom expression [31,63,70]. In a large ASD + OCD sample, Leyfer et al. found that the most common type of compulsion was a ritual involving other individuals having to perform a certain way (e.g., greeting and separation rituals, reassurance-seeking rituals) [31]. On the other hand, youth with ASD + OCD were significantly less likely to endorse sexual obsessions (F = 4.3; *p* = 0.04), checking rituals (F = 10.2; *p* = 0.002), washing (F = 8.2; *p* = 0.006), or repeating compulsions (F = 4.2; *p* = 0.04) than their counterparts without ASD [63]. Mack et al. reported no significant differences between OCD-only and OCD/ASD paediatric samples in a small study. However, they noted that patients meeting criteria for both diagnoses reported higher functional impairment, compared with children with OCD only [70]. Several studies also noted the difficulties in differentiating the differential diagnosis of OCD-like behaviours among children with ASD and adolescent samples from ASD-related behaviours [43,46,50], either because the parents or guardians did not know if rituals caused distress to the children [40], or if the obsessional thoughts and compulsions had a purposeful quality [41], both of which are diagnostic criteria of obsessive compulsive disorder.

### 3.5. Prognostic Factors

Another set of six studies assessed the severity and prognostic implications of the concurrence of both disorders. OCD and ASD co-occurrence was associated with significantly higher functional impairment [57,62,67], poorer insight regarding their OCD [67], greater levels of concurrent psychopathology [67], and overall comorbidities [63], compared to youth with OCD but without comorbid ASD. Reversely, comorbid OCD was a significant risk factor for overall symptom severity and psychiatric hospitalization among children with ASD [50,56]. On the other hand, Lewin et al. found, in a small case–control study, that patients with OCD and concurrent ASD did not differ from patients with OCD for total severity scores, obsessions, nor compulsion scores [63].

Two additional studies studied the impact of both disorders on families of youth with OCD + ASD, and both found out that families of children and adolescents with both OCD and ASD engaged in more accommodating behaviours [62,67].

### 3.6. Therapeutic Factors

Several studies assessed the therapeutic implications of OCD and ASD concurrence among children and adolescents. In terms of service utilization, Martin et al. found in a large cohort that youth with OCD and ASD were more likely to be prescribed medication and use health services for longer than either OCD-only or ASD-only children, with significant improvement after health service utilization but with smaller gains than those children who had OCD without ASD [57].

Nine studies assessed the efficacy of cognitive behavioural therapy (CBT) for samples of youth with concurrent OCD and ASD. Three of them offered patients CBT protocols specially adapted for people with ASD, either face-to-face [66,69] or internet-delivered [65]. All these studies showed these modified protocols were acceptable and associated with clinically significant improvements in CY-BOCS scores and OCD-like behaviours, although in-person CBT seemed to perform significantly better. One additional study offered patients intensive CBT (3–6 h/day for up to 80 sessions) with ASD-specific modifications, obtaining a response rate of 78%, with large treatment effects in OCD symptom severity [49]. Five additional studies administered patients unmodified CBT protocols. Except for Højgaard et al., who did not find significant differences in treatment outcomes of OCD patients with or without concurrent ASD traits [68], all of them showed significantly lower rates of treatment response (38.3% vs. 48.2%) and remission (9.0% vs. 46.0%) [54,62,64,67] for youth in the OCD + ASD groups.

On the other hand, there were no studies specifically studying the efficacy of pharmacological treatments on OCD among children and adolescents with ASD. However, King et al. studied the efficacy of citalopram in children with ASD and high levels of repetitive behaviour (scoring at least moderate on compulsive behaviours measured with the Children’s Yale–Brown Obsessive Compulsive Scales, CY-BOCS), and found no significant difference in the rate of positive response on the clinical global impression nor in score reduction on the CY-BOCS, compared to the placebo (REF). Furthermore, citalopram use was significantly more likely to be associated with adverse effects [48]. Schachar et al. found that antipsychotic use was uncommon among OCD cases without a comorbidity (1–2%) but was significantly more common among those with concurrent ASD (9–19%), although no study formally assessed the efficacy or safety of these compounds on children and adolescents with the comorbidity [52].

### 3.7. Quality of the Included Studies

The mean NOS score for the included studies was 6.78 ± 0.90. Of the included studies, 59.3% and 40.74% had a low and moderate risk of bias, respectively (Table 1 and Table 2). The most common cause for bias among the included studies was the potential lack of representativeness of the samples, as most studies were based on clinical samples, which may constitute a referral bias [72].

## 4. Discussion

To the best of our knowledge, this is the first study to provide a comprehensive view of the current status of knowledge about the concurrence between obsessive compulsive disorder and autism spectrum disorder among children and adolescents. It includes meta-analytical evidence on its prevalence, along with a systematic review on its clinical and therapeutical implications.

Several important findings arise from it. OCD is highly prevalent among youth with ASD, with a pooled prevalence of 11.6%, which represents a five to six-fold increase relative to the 2% prevalence of OCD reported in the general paediatric population [73]. Furthermore, several studies stress that clinically significant obsessive and compulsive symptoms are common among those children and adolescents with ASD who do not meet OCD criteria [17,34,39,40,46]. Our results show that ASD is also common among youth with OCD, with a pooled prevalence of 11.5%. This represents a substantially elevated prevalence of ASD in youth with OCD, compared to the population prevalence rate of ASD in children and adolescents, which is estimated to be 1–3% [74]. There is also evidence that a substantial proportion of youth with OCD show moderate ASD traits that do not meet the full diagnostic threshold for ASD but that may nevertheless be clinically important [56,62]. It must be noted, however, that almost all of the included studies in our meta-analyses were based on clinical populations instead of community-based samples. Since clinical samples may not be representative of the general population, due to biases such as referral and Berkson bias, our results are probably most relevant to help-seeking individuals.

There are several possible explanations for the high rate of concurrence between ASD and OCD. One possibility is phenomenological overlap which may artificially inflate estimates of co-occurrence. Many obsessive compulsive symptoms can present similarly to the core features of ASD and vice versa, such as obsessive ideation and restrictive interests, or compulsive behaviours and stereotypies. This, as several studies included in our systematic review note [40,41,46], implies significant challenges when stablishing differential diagnoses. It is also important to note that included studies reported a wide range of concurrent prevalence, from 0.6% [35] to 55.0% [29]. We believe this may reflect to some degree a misclassification of restrictive, repetitive behaviours and interests as connoting OCD symptoms, and vice versa. For instance, electronic insurance health records and other similar registers where there is no individual assessment of the comorbid and concurrent disorders might not be the best data sources for estimating the real prevalence of comorbidities, due to some level of under-diagnosis [75]. This is particularly true for ASD, where intellectual disability may hinder accurate assessment of overlapping symptoms of co-occurring disorders [76].

On the other hand, there is growing evidence of common etiopathogenetic factors between the two disorders, which could explain genuine comorbidity between ASD and OCD. Family studies have found both higher OCD symptoms among first-grade relatives of children with ASD [77], and significant ASD traits among parents of children with OCD [78], and genes linked to OCD and ASD present significant overlap, with potential interactions between them [79,80,81]. There is some evidence that this could be particularly significant for a subset of ASD patients, where OCD symptoms (even when the complete criteria for the diagnosis are not met) define a distinct phenotype of the disorder [82]. Moreover, autism, defined as a neurodevelopmental disorder, represents a risk factor or vulnerability state for various mental health difficulties, including challenges in dealing with anxiety and exhibiting flexibility in appropriate responses, thereby paving the way for maladaptive responses such as compulsive behaviours.

The high rate of concurrence between autistic and OCD behaviours can also be explained by certain theoretical approaches. Present nosological systems are the result of a consensus among experts on the most appropriate way to classify various forms of human dysfunction, based on several criteria and decisions, which directly impact comorbidity prevalence rates. Therefore, they are not supposed to reflect natural distinct entities but rather serve as frameworks for understanding.

Two risk factors for being diagnosed with both OCD and ASD have been identified: sex and age. Three studies found that male children and adolescents with OCD were significantly more likely to meet criteria for ASD than females [51,52,55], which is also supported by the meta-regressions in our study. This aligns with previous reports in the literature, with ASD showing a male preponderance with a sex ratio of 4.3:1 [83]. Another study, however, found higher rates of OCD prevalence among male than female children with ASD [32], while, in the general population, women have a higher lifetime risk of presenting OCD [84]. This inconsistency may be specific to paediatric populations and can be attributed by the age-of-onset of OCD, with males making up the majority of early-onset cases, specially before the age of 10 [85]. Additionally, two studies found older age to be a risk factor for OCD among ASD samples [35,36], as this disorder tends to onset during adolescence or young adulthood. This contrasts with the findings in our study, where the meta-regression performed with the mean age of the sample was not statistically significant, probably due to a lack of statistical power. In the clinical setting, these findings imply that clinicians should be especially vigilant for this concurrence in male adolescent patients being treated for either ASD or OCD.

A few studies assessed the existence of distinct patterns of symptom presentation among children with OCD and ASD. Autistic youth with OCD exhibit fewer sexual and magical obsessions, washing, and repeating compulsions than children with only OCD [63], along with higher levels of hoarding and ordering behaviours than children and adolescents without ASD, along with rituals involving other people [31]. Mack et al. did not find significant different in terms of symptom expression among two small samples of OCD-only and OCD/ASD children. However, they did find higher functional impairment in the latter group [70]. In connection to this, two studies found higher levels of family accommodation to OCD-related symptoms in families with autistic children [62,67]. However, the most repeated finding in terms of symptom expression was the difficulties in differentiating OCD-like behaviours from ASD-related behaviours. Some specific tools for this population, such as the CY-BOCS adapted for ASD population [86], would help future lines of research. Some evidence was found as well for the prognostic implications of the dual diagnosis among children and adolescents, although this remains limited due to the cross-sectional nature of the studies. Except for one small study [63], OCD and ASD concurrence was vastly associated with higher functional impairment, poorer insight regarding symptoms, and greater levels of psychopathology and comorbidities, compared to youth with either of the disorders alone. However, the lifelong trajectories of these children and adolescents, along with protective and risk factors for impaired functioning at adult age, remain unclear due to the absence of longitudinal research.

As for the therapeutical implications of OCD and ASD concurrence, we found substantial evidence supporting the efficacy of CBT in this population, even though some points need to be addressed. While several studies reported positive outcomes of standard CBT protocols on youth with OCD, all of them found that those with ASD responded significantly less well to treatment (including lower mean change scores on CY-BOCS, along with lower response and remission rates) than their counterparts without ASD [51,52,54,55,62,64]. Højgaard et al., on the other hand, found that CBT for OCD was equally effective for those with and without ASD traits; however, in that particular study, autistic traits as a continuum were studied, instead of criteria-meeting ASD, which could explain this inconsistency [68]. Taken together, findings indicate that CBT is beneficial for children and adolescents with OCD + ASD, but that standard protocols may not be as effective as in those with neurotypical development. Many young people with ASD struggle when identifying thoughts and emotions [87], along with planning and organizing tasks and considering alternative solutions or cognitions [88], all of which are key components of standard CBT protocols. Hence, tailored protocols must be developed for this population, considering adaptive skills deficits and limited insight, among other factors. Some studies reported the use of modified CBT protocols for children with ASD and adolescents. One small, preliminary RCT introduced function-based behavioural assessment components to the standard CBT protocol, obtaining significantly decreased obsessive compulsive behaviours in pre-adolescents with both OCD and ASD [69], while another naturalistic, small study obtained high rates of response and remission with an ASD-adapted CBT manual for adolescents, maintained at a 3-month follow-up [66]. Another feasibility uncontrolled study provided intensive, internet-delivered, and modified CBT to the same population, also obtaining promising results with a large effect size (*d* = 1.33), even though in-person CBT produced significantly greater effects (*d* = 2.69) [65]. Interestingly, a recent meta-analysis found that remotely delivered CBT appears efficacious in reducing OCD symptoms, but individuals with severe symptomatology may benefit more from face-to-face than remote CBT [89]. However, this evidence remains highly preliminary, and future research would benefit from conducting larger, controlled RCTs of psychotherapeutic interventions in this population.

Another important finding of this review was the scarcity of studies assessing the efficacy of pharmacological interventions on the paediatric population with concurrent OCD and ASD. This is particularly important, considering that the combination of CBT and selective serotonin reuptake inhibitors (SSRIs) remains the gold standard for moderate to severe OCD in children [90]. One randomized clinical trial suggested that citalopram was not superior to placebo in the reduction in CY-BOCS score among a youth sample with ASD [48]. Another research suggested antipsychotics are used more frequently to treat OCD in children with ASD compared to those without [52]. While there is extensive evidence that aripiprazole and risperidone are significantly better than a placebo, both at improving emotional dysregulation and irritability in ASD [91] and as an augmentation strategy for SSRI-resistant OCD [92], this must be taken with caution, as children with ASD are particularly prone to suffer adverse effects of medications [93]. This stresses the need of clinicians to rely on scientific evidence to select pharmacological treatments for this population. Future research should focus on conducting randomized clinical trials to assess the efficacy and tolerability of psychopharmacological interventions in general, and SSRIs and antipsychotics in particular, in the treatment of paediatric concurrent OCD and ASD.

This is the first study to meta-analytically assess both the prevalence of OCD among ASD paediatric samples and the ASD prevalence among OCD samples of children and adolescents, along with its clinical and therapeutical implications. This offers a broad perspective on this concurrence, along with valuable insight and hints on the directions future research on the topic should take. Notwithstanding all the above, some important limitations of our research must be noted. First, most of the included studies were based on clinical samples, which as discussed above, may constitute a referral bias. Second, considerable heterogeneity was found for both meta-analytical outcomes. Some authors have used different scales and diagnostic criteria for both OCD and ASD, and the included samples were significantly heterogeneous in terms of symptom severity and age. Third, our findings might not be globally representative, as almost all the included studies were conducted either in Europe or in North America. Future studies would benefit from the study of this concurrence and its implications in alternative countries and cultural contexts. Fourth, our findings on the prognostic implications of OCD and ASD concurrence among youth is limited by the cross-sectional nature of almost all included studies. Finally, most of the studies did not include a control group of typically developing, healthy children, so a comparison with general population prevalence could not be provided.

## 5. Conclusions

Our results indicate that ASD and OCD are commonly concurrent disorders among children and adolescents, with a co-occurrence prevalence of over 11.0% for both conditions. Differentiating the two disorders can be a diagnostic challenge but has important clinical implications. Children and adolescents with dual diagnosis present with higher rates of functional impairment, psychopathology, and other comorbidities, compared to youth with either of the disorders alone, clinicians should be especially vigilant for concurrent ASD and OCD among male adolescent patients. Although available evidence favours the use of ASD-adapted CBT protocols in the treatment of OCD in this population, there is a need for large RCTs examining the efficacy of psychotherapeutic and pharmacological interventions. Future research should focus on conducting longitudinal cohort studies in order to understand prognostic factors, determine development trajectories among this population and clarify the co-occurrence implications at every developmental stage.

## Data Availability

The raw data supporting the conclusions of this article will be made available by the authors upon reasonable request. The data are not publicly available because of privacy concerns.

## Supplementary Materials

The following supporting information can be downloaded at: https://www.mdpi.com/article/10.3390/brainsci14040379/s1, Table S1: PRISMA statement and checklist; Table S2: MOOSE Statement checklist; Table S3: Quality assessment: Newcastle-Ottawa Scale (NOS) for Cohort Studies; Table S4: Meta-regressions for the OCD prevalence among ASD samples; Table S5: Subgroup analyses for the OCD prevalence among ASD samples; Table S6: Meta-regressions for the ASD prevalence among OCD samples; Figure S1: Funnel plot for publication bias for the OCD prevalence among ASD samples; Figure S2: Funnel plot for publication bias for the ASD prevalence among OCD samples.
