# Supplementary material for: Prevalence and Correlates of the Concurrence of Autism Spectrum Disorder and Obsessive Compulsive Disorder in Children and Adolescents: A Systematic Review and Meta-Analysis
Source: Brain Sci. 2024 Apr 13;14(4):379. doi: 10.3390/brainsci14040379 (PMC11048346; doi:10.3390/brainsci14040379)

SUPPLEMENTARY MATERIAL

Table S1: PRISMA statement and checklist……………………………………………………………………………….................page 2-3

Table S2: MOOSE Statement checklist……………………..……………………………………………………………………….page 4-5

Table S3: Quality assessment: Newcastle-Ottawa Scale (NOS) for Cohort Studies…………………………………………………...…....page 6

Table S4: Meta-regressions for the OCD prevalence among ASD samples………..…………………………………………….…...….page 7

Table S5: Subgroup analyses for the OCD prevalence among ASD samples……………………………..………..…………….…...….page 8

Table S6: Meta-regressions for the ASD prevalence among OCD samples………..…………………………………………….….….page 9

Figure S1: Funnel plot for publication bias for the OCD prevalence among ASD samples……..……………………………….……….….page 10

Figure S2: Funnel plot for publication bias for the ASD prevalence among OCD samples……..…………………………….……….……..page 11

Table S1: PRISMA statement and checklist.

Section/Topic	Item #	Checklist item	Page
**TITLE**			
Title	1	Identify the report as a systematic review.	1
**ABSTRACT**			
Abstract	2	Provide a structured summary including, as applicable: background; objectives; data sources; study eligibility criteria, participants, and interventions; study appraisal and synthesis methods; results; limitations; conclusions and implications of key findings; systematic review registration number.	2
**INTRODUCTION**			
Rationale	3	Describe the rationale for the review in the context of existing knowledge.	3
Objectives	4	Provide an explicit statement of the objective(s) or question(s) the review addresses.	3
**METHODS**			
Eligibility criteria	5	Specify the inclusion and exclusion criteria for the review and how studies were grouped for the syntheses.	4
Information sources	6	Specify all databases, registers, websites, organisations, reference lists and other sources searched or consulted to identify studies. Specify the date when each source was last searched or consulted.	4
Search strategy	7	Present the full search strategies for all databases, registers and websites, including any filters and limits used.	4
Selection process	8	Specify the methods used to decide whether a study met the inclusion criteria of the review, including how many reviewers screened each record and each report retrieved, whether they worked independently, and if applicable, details of automation tools used in the process.	5
Data collection process	9	Specify the methods used to collect data from reports, including how many reviewers collected data from each report, whether they worked independently, any processes for obtaining or confirming data from study investigators, and if applicable, details of automation tools used in the process.	5
Data items	10	List and define all outcomes for which data were sought and if any assumptions were made about any missing or unclear information.	5
Study risk of bias assessment	11	Specify the methods used to assess risk of bias in the included studies, including details of the tool(s) used, how many reviewers assessed each study and whether they worked independently, and if applicable, details of automation tools used in the process.	5
Effect measures	12	Specify for each outcome the effect measure(s) (e.g. risk ratio, mean difference) used in the synthesis or presentation of results.	6
Synthesis methods	13	Describe the processes used to decide which studies were eligible for each synthesis. Describe any methods required to prepare the data for presentation or synthesis, such as handling of missing summary statistics, or data conversions. Describe any methods used to tabulate or visually display results of individual studies and syntheses. Describe any methods used to synthesize results and provide a rationale for the choice(s). Describe the model(s), method(s) to identify the presence and extent of statistical heterogeneity, and software package(s) used, any methods used to explore possible causes of heterogeneity among study results (e.g. subgroup analysis, meta-regression) and any sensitivity analyses conducted to assess robustness of the synthesized results.	6
Reporting bias assessment	14	Describe any methods used to assess risk of bias due to missing results in a synthesis (arising from reporting biases).	6

Certainty assessment	15	Describe any methods used to assess certainty (or confidence) in the body of evidence for an outcome.	6
**RESULTS**			
Study selection	16	Describe the results of the search and selection process, from the number of records identified in the search to the number of studies included in the review, ideally using a flow diagram. Cite studies that might appear to meet the inclusion criteria, but which were excluded, and explain why they were excluded.	6
Study characteristics	17	Cite each included study and present its characteristics.	19-24
Risk of bias in studies	18	Present assessments of risk of bias for each included study.	19-24
Results of individual studies	19	For all outcomes, present, for each study: (a) summary statistics for each group (where appropriate) and (b) an effect estimate and its precision (e.g. confidence/credible interval), ideally using structured tables or plots.	19-24
Results of syntheses	20	For each synthesis, briefly summarise the characteristics and risk of bias among contributing studies. Present results of all statistical syntheses conducted. If meta-analysis was done, present for each the summary estimate and its precision (e.g. confidence/credible interval) and measures of statistical heterogeneity. If comparing groups, describe the direction of the effect. Present results of all investigations of possible causes of heterogeneity among study results and all sensitivity analyses conducted to assess the robustness of the synthesized results.	7
Reporting biases	21	Present assessments of risk of bias due to missing results (arising from reporting biases) for each synthesis assessed.	10, Supps
Certainty of evidence	22	Present assessments of certainty (or confidence) in the body of evidence for each outcome assessed.	19-24
**DISCUSSION**			
Discussion	23	Provide a general interpretation of the results in the context of other evidence. Discuss any limitations of the evidence included in the review, any limitations of the review processes used and the implications of the results for practice, policy, and future research.	10-14
**OTHER INFORMATION**			
Registration and protocol	24	Provide registration information for the review, including register name and registration number, or state that the review was not registered. Indicate where the review protocol can be accessed, or state that a protocol was not prepared. Describe and explain any amendments to information provided at registration or in the protocol.	4
Support	25	Describe sources of financial or non-financial support for the review, and the role of the funders or sponsors in the review.	14
Competing interests	26	Declare any competing interests of review authors.	14
Availability of data, code and other materials	27	Report which of the following are publicly available and where they can be found: template data collection forms; data extracted from included studies; data used for all analyses; analytic code; any other materials used in the review.	14

Table S2: MOOSE Statement - Reporting Checklist for Authors, Editors, and Reviewers of Meta-analyses of Observational Studies

Reporting Criteria	Reported (Yes/No)	Reported on Page
**Reporting of Background**		
Problem definition	Yes	3
Hypothesis statement	Yes	3
Description of Study Outcome(s)	Yes	3
Type of exposure or intervention used	Yes	3
Type of study design used	Yes	3
Study population	Yes	3
**Reporting of Search Strategy**		
Qualifications of searchers (eg, librarians and investigators)	Yes	4
Search strategy, including time period included in the synthesis and keywords	Yes	4
Effort to include all available studies, including contact with authors	Yes	4
Databases and registries searched	Yes	4
Search software used, name and version, including special features used (eg, explosion)	N.a.	4
Use of hand searching (eg, reference lists of obtained articles)	Yes	4
List of citations located and those excluded, including justification	Yes	15, 19-24
Method for addressing articles published in languages other than English	Yes	4
Method of handling abstracts and unpublished studies	Yes	4
Description of any contact with authors	N.a.	4
**Reporting of Methods**		
Description of relevance or appropriateness of studies assembled for assessing the hypothesis to be tested	Yes	5
Rationale for the selection and coding of data (eg, sound clinical principles or convenience)	Yes	5
Documentation of how data were classified and coded (eg, multiple raters, blinding, and interrater reliability)	Yes	5
Assessment of confounding (eg, comparability of cases and controls in studies where appropriate)	Yes	5
Assessment of study quality, including blinding of quality assessors; stratification or regression on possible predictors of study results YES 5	Yes	5
Assessment of heterogeneity	Yes	5
Description of statistical methods (eg, complete description of fixed or random effects models, justification of whether the chosen models account for predictors of study results, dose-response models, or cumulative meta-analysis) in sufficient detail to be replicated	Yes	5-6
Provision of appropriate tables and graphics	Yes	16-24
**Reporting of Results**		
Table giving descriptive information for each study included	Yes	19-24
Results of sensitivity testing (eg, subgroup analysis)	Yes	7
Indication of statistical uncertainty of findings	Yes	7

**Reporting of Discussion**		
Quantitative assessment of bias (eg, publication bias)	Yes	Supps
Justification for exclusion (eg, exclusion of non–English-language citations)	Yes	15
Assessment of quality of included studies	Yes	7
**Reporting of Conclusions**		
Consideration of alternative explanations for observed results	Yes	10-14
Generalization of the conclusions (ie, appropriate for the data presented and within the domain of the literature review)	Yes	10-14
Guidelines for future research	Yes	10-14
Disclosure of funding source	Yes	14

Table S3: Quality assessment: Newcastle-Ottawa Scale (NOS) for Cohort Studies

Quality of the included studies was assessed using the Newcastle-Ottawa Scale (NOS) for Cohort Studies due to the heterogeneity expected in the included studies. The following assessment scores were used:

Criteria	Maximum Score
Representativeness of exposed cohort	★
Selection of the non-exposed cohort	★
Ascertainment of exposure	★
Demonstration that outcome of interest was not present at start of study	★
Comparability of cohorts based on the design or analysis controlled for confounders	★ ★
Assessment of outcome	★
Was follow-up long enough for outcomes to occur	★
Adequacy of follow-up of cohorts	★

Table S4: Meta-regressions for the OCD prevalence among ASD samples.

Meta-regression	No. of Studies	β Coefficient	SE	95% CI		P value
**% Female**	20	-0.0312	0.240	-0.535	0.473	0.90
**IQ Mean**	14	-0.0321	0.025	-0.087	0.023	0.23
**NOS Score**	21	-0.0235	0.291	-0.633	0.586	0.94
**Mean age**	21	0.0915	0.178	-0.281	0.464	0.61
**Year of publication**	21	-0.0690	0.039	-0.150	0.012	0.09

CI Confidence Interval; SE Standard Error; IQ Intelligence Quotient; NOS Newcastle-Ottawa Scale.

Table S5: Subgroup analyses for the OCD prevalence among ASD samples.

	No. Studies	Sample size	Proportion	95% CI	Heterogeneity	
					I^2 (%)	*p*
**Continent**	Test for between groups difference: Q = 40.69; p < 0.001*					
Europe	14	8283	0.0991	0.0525 – 0.1793	95.7	<0.01
North America	6	573	0.1208	0.0418 – 0.3019	88.1	<0.01
**Diagnostic Criteria**	Test for between groups difference: Q = 0.53; p = 0.47					
DSM	18	1984	0.1189	0.0656 – 0.2061	91.6	< 0.01
ICD	3	6932	0.0838	0.0138 – 0.3739	87.1	0.01
**ASD Sample**	Test for between groups difference: Q = 0.42; p = 0.52					
Only ASD	11	8158	0.0994	0.0408 – 0.2227	97.2	< 0.01
ASD + PDD	10	758	0.1355	0.0690 – 0.2489	91.7	< 0.01

CI Confidence Interval; DSM Diagnostic and Statistical Manual of Mental Diseases; ICD International Classification of Diseases; ASD Autism Spectrum Disorder; PDD Pervasive Developmental Disorder.

Table S6: Meta-regressions for the ASD prevalence among OCD samples.

Meta-regression	No. of Studies	β Coefficient	SE	95% CI		P value
**% Female**	11	-4.6894	1.992	-8.593	-0.786	0.02*
**Mean age**	10	-0.0388	0.259	-0.547	0.469	0.88
**NOS Score**	11	0.2036	0.309	-0.402	0.809	0.66
**Year of publication**	11	-0.021	0.065	-0.149	0.107	0.74

CI Confidence Interval; SE Standard Error; IQ Intelligence Quotient; NOS Newcastle-Ottawa Scale.

Figure S1: Funnel plot for publication bias for the OCD prevalence among ASD samples.

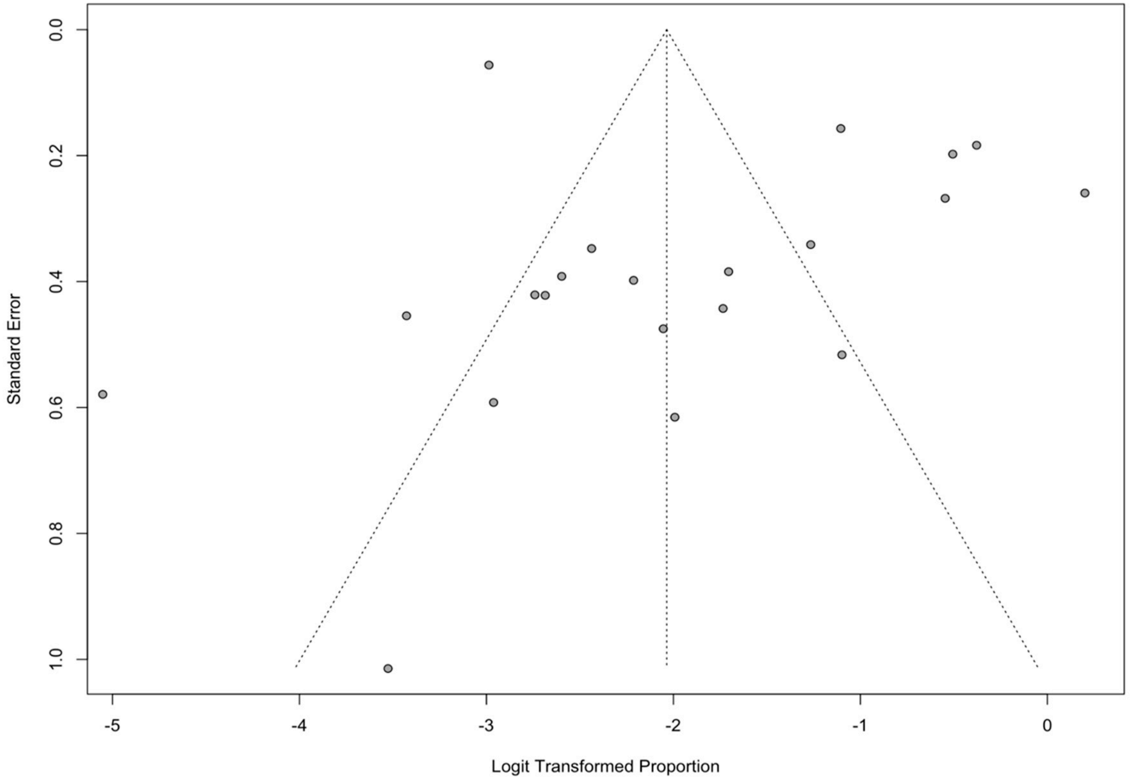

Figure S2: Funnel plot for publication bias for the ASD prevalence among OCD samples.

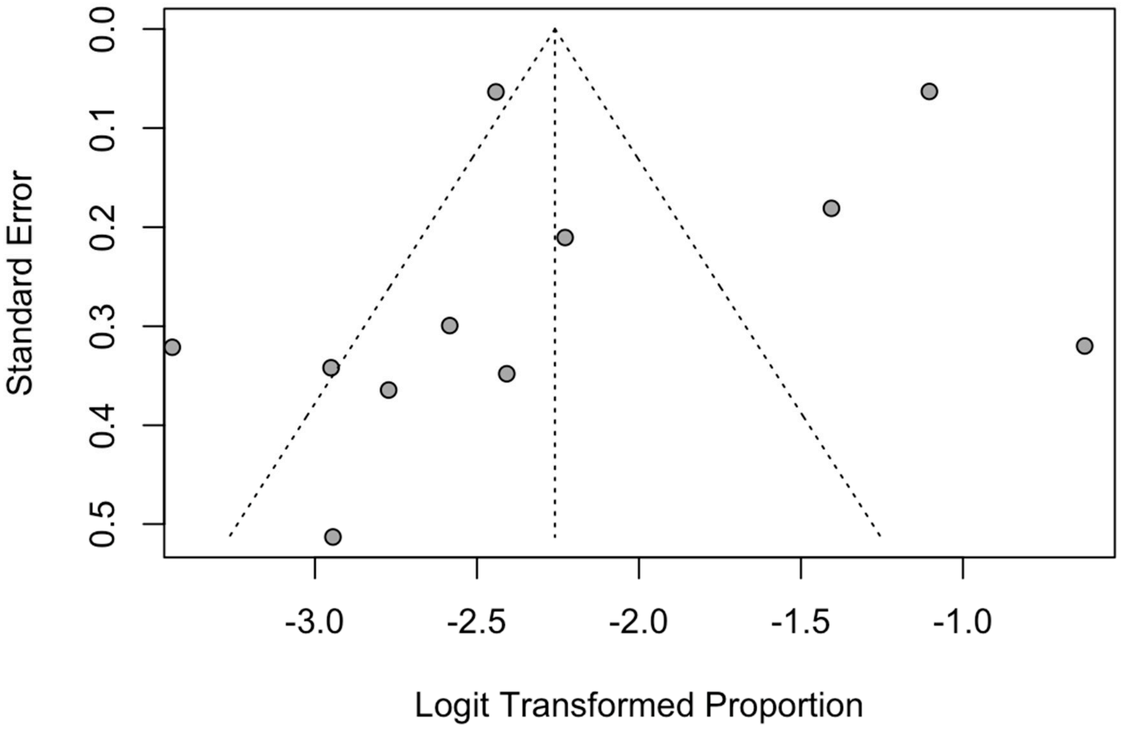

Supplement: Supplementary file 1 [file brainsci-14-00379-s001.zip › brainsci-2946370-supplementary.pdf]
